# Supplementary material for: RGS14 is neuroprotective against seizure-induced mitochondrial oxidative stress and pathology in hippocampus
Source: bioRxiv. 2023 Feb 3:2023.02.01.526349. Preprint. [Version 1] doi: 10.1101/2023.02.01.526349 (PMC9915580; doi:10.1101/2023.02.01.526349)
Supplement: Supplement 1 [file media-1.pdf]

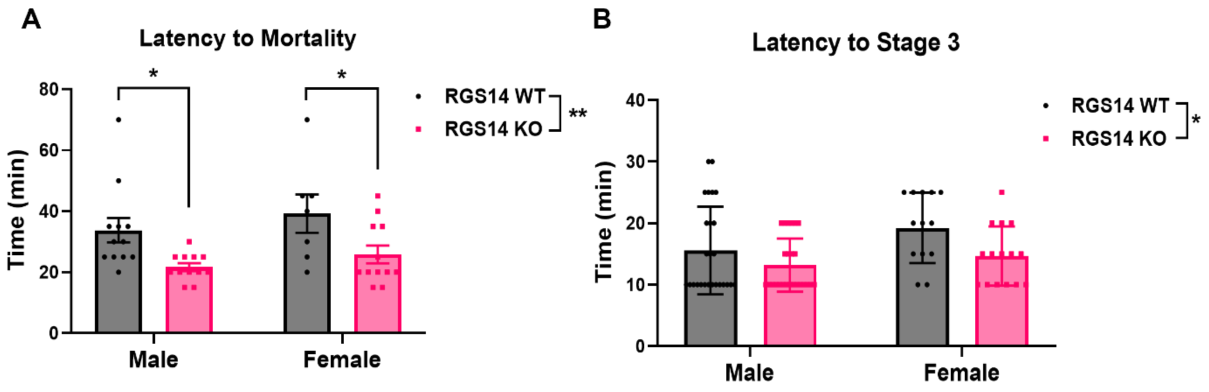

Supplemental Figure S1. Behavioral sensitivity to KA-SE in RGS14 KO mice is not affected by sex. (A) Mean latency to mortality in male and female WT and RGS14 KO mice (males: WT, 33.75 ± 4.00 min; RGS14 KO, 21.67 ± 1.28 min) (females: WT, 39.29 ± 6.31 min; RGS14 KO, 25.83 ± 2.94 min). (B) Mean latency to Stage 3 behavioral seizure activity in male and female WT and RGS14 KO mice. (A, B) Two-ANOVA with Sidak post-hoc comparisons was used to compare mean latency to mortality (A; two-way ANOVA, F = 12.60 for genotype, **p < 0.01; Sidak, WT vs RGS14 KO, *p < 0.05) and Stage 3 seizure activity (two-way ANOVA, F = 6.71 for genotype, *p<0.05). Error bars represent the SEM.

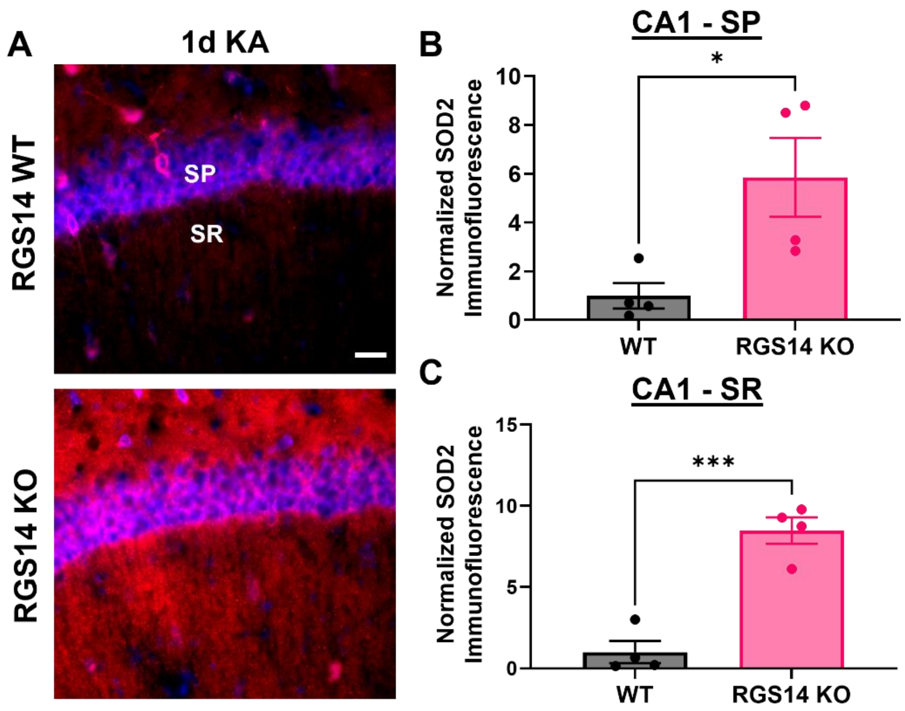

Supplemental Figure S2. SOD2 is upregulated in area CA1 of RGS14 KO mice one day after KA-SE. (A) Representative IHC images of superoxide dismutase 2 (SOD2) expression in WT and RGS14 KO CA2 one day after KA-SE. SP and SR represent regions of analysis for cell body (SP) and dendritic (SR) layers of CA2. (B) Mean SOD2 immunofluorescence in the SP layer of CA1 expressed relative to WT mean (WT, 1.00 ± 0.52; RGS14 KO, 5.85 ± 1.62). (C) Mean SOD2 immunofluorescence in the SR layer of CA1 expressed relative to WT mean (WT, 1.00 ± 0.68; RGS14 KO, 8.48 ± 0.81). (B, C) Group means compared using unpaired t-tests (B, WT vs RGS14 KO, *$p < 0.05$) (C, WT, vs RGS14 KO, ***$p < 0.001$). Error bars represent the SEM. SP, stratum pyramidale; SR, stratum radiatum.

A **SAL**

Upregulated in KO

Protein	Adj. p-value	Log2 FC (KO/WT)
Ehbp1	0.00342	1.848
Ppfia4	0.00146	0.707
Them6	0.01911	0.537
Ocrl	0.01824	0.518
Abcb1a	0.00534	0.381
Ndufaf4	0.03588	0.309
Sh3kbp1	0.02090	0.290
Atp5f1c	0.03784	0.167

Downregulated in KO

Protein	Adj. p-value	Log2 FC (KO/WT)
Rgs14	0.00003	-4.239
Tpm1	0.04297	-1.405
Spag7	0.00010	-1.054
Gfer	0.00540	-0.907
Uqcr11	0.01041	-0.899
Rap2c	0.00878	-0.808
Nfix	0.02050	-0.779
Pigu	0.03585	-0.626
Fto	0.01307	-0.597
Syt11	0.04393	-0.591
Naa15	0.01176	-0.579
Trappc8	0.02520	-0.537
Naaa	0.02662	-0.521
Ggact	0.04601	-0.517
Psap	0.03392	-0.501
Srrm2	0.03583	-0.496
Ndrg4	0.04098	-0.483
Rab9a	0.03169	-0.472
Atp5md	0.04234	-0.448
Tiprl	0.00271	-0.420
Ddost	0.01995	-0.418
Scg5	0.03750	-0.401
Edc4	0.03111	-0.395
Erlin2	0.00245	-0.323
Akt1	0.01005	-0.322
Agfg2	0.01151	-0.319
Scn1b	0.04604	-0.310
Dynll1	0.04879	-0.277
Alcam	0.04788	-0.263
Ggt7	0.04351	-0.252
G6pdx	0.02414	-0.245
Slc4a4	0.04152	-0.190
Ndufv1	0.03694	-0.151
Gnas	0.02256	-0.139
Cct5	0.04734	-0.127
Actn1	0.01044	-0.097
Fasn	0.01851	-0.087

B **KA**

Upregulated in KO

Protein	Adj. p-value	Log2 FC (KO/WT)
Apob	0.03976	1.116
Ehbp1	0.01315	0.930
Txndc12	0.01455	0.825
Bckdhb	0.04040	0.796
Mlst8	0.01556	0.731
Ppfia4	0.01394	0.538
Arsb	0.00106	0.488
Cygb	0.03770	0.477
Tmed7	0.02162	0.477
Trappc8	0.04369	0.452
Dlgap2	0.03178	0.419
Acat2	0.02662	0.385
Sod2	0.02007	0.329
Sort1	0.03027	0.303
Ssr1	0.01621	0.293
Eno1	0.03296	0.090

Downregulated in KO

Protein	Adj. p-value	Log2 FC (KO/WT)
Rgs14	0.00005	-4.070
Sf3b1	0.03869	-0.904
Gfer	0.03674	-0.851
Fto	0.00107	-0.808
Hnrnph3	0.03684	-0.690
Srgap2	0.04268	-0.654
Prrc2c	0.01850	-0.647
Gabbr1	0.04053	-0.637
Elmo1	0.01756	-0.627
Stx8	0.02297	-0.551
Hagh	0.00063	-0.517
C2cd4c	0.03492	-0.508
Trappc13	0.01821	-0.494
Anxa3	0.03948	-0.483
Cacna1e	0.01062	-0.472
Ptgr2	0.01220	-0.460
Rgs7bp	0.00155	-0.459
Ubr4	0.01557	-0.451
Bola2	0.04830	-0.414
Dgke	0.04097	-0.398
Esd	0.03030	-0.368
Mpp3	0.01045	-0.311
Acaa1a	0.02432	-0.306
Asah1	0.02940	-0.303
Npc1	0.01759	-0.297
Psmc6	0.01991	-0.292
Tiprl	0.04249	-0.282
Ncam1	0.04696	-0.282
Sh3kbp1	0.03674	-0.265
Hdhd2	0.01715	-0.257
Spr	0.04502	-0.158
Actr1b	0.01800	-0.153
Fasn	0.00172	-0.119

Supplemental Table 1. Comprehensive list of differentially expressed proteins (DEPs) between WT and RGS14 KO after SAL or KA treatment. (A) List of upregulated (left) or downregulated (right) DEPs in RGS14 KO hippocampi one day after saline treatment, their adjusted p-values, and the fold change of abundance (log_2 FC (KO/WT)). (B) List of upregulated (left) or downregulated (right) DEPs in RGS14 KO hippocampi one day after saline treatment, their adjusted p-values, and the fold change of abundance (log_2 FC (KO/WT)). FC, fold change.

A **WT**

Upregulated after KA

Protein	Adj. p-value	Log2 FC (KA/Sal)
Lrrfip1	0.04855	2.039
Hspb1	0.01937	1.367
Dnajb5	0.00046	1.217
Ybx1	0.03460	1.132
Vgf	0.00210	0.952
Castor2	0.04962	0.932
Lsm3	0.03750	0.927
Ptpn5	0.04391	0.643
Rps27	0.01114	0.608
Psmb5	0.04123	0.500
Fmnl1	0.02409	0.491
Prmt1	0.03650	0.473
Ndufaf4	0.00109	0.473
Ddi2	0.03225	0.456
Btf3	0.02712	0.434
Spon1	0.02731	0.433
Rab8a	0.02410	0.397
Eif1	0.01400	0.379
G3bp2	0.02647	0.341
Tpt1	0.00006	0.334
Hsph1	0.00032	0.324
Sh3kbp1	0.01395	0.291
Rps16	0.00703	0.290
Dnaja1	0.01025	0.280
Grb2	0.01957	0.252
Eef1a1	0.03566	0.235
Eef2	0.00045	0.221
Tra2a	0.01602	0.215
Rheb	0.04696	0.214
Kiaa0513	0.03774	0.206
Rpl13	0.03803	0.205
Hsp90ab1	0.02585	0.195
Hpcal4	0.00062	0.191
Hspa8	0.03850	0.131

Downregulated after KA

Protein	Adj. p-value	Log2 FC (KA/Sal)
Bckdhb	0.02584	-0.810
Tinagl1	0.04738	-0.796
Kcnj10	0.02556	-0.758
Naa15	0.00127	-0.722
Ank1	0.01913	-0.649
Abcd3	0.00731	-0.624
Snrpa1	0.01207	-0.561
Ggact	0.02672	-0.535
Aldh4a1	0.02659	-0.529
Hectd4	0.00104	-0.508
Cygb	0.02392	-0.486
Trappc8	0.02779	-0.440
Scg5	0.01695	-0.430
Ddhd1	0.01846	-0.393
Pnmal2	0.04917	-0.356
Akt1	0.00585	-0.353
C20orf27	0.02376	-0.352
Tiprl	0.00950	-0.337
Erlin2	0.00276	-0.300
Hadha	0.00548	-0.300
Sorbs1	0.04445	-0.282
Hadhb	0.04115	-0.277
Alcam	0.03304	-0.265
Scfd1	0.01639	-0.265
Pgls	0.02616	-0.262
Hnrnpr	0.02022	-0.253
Dgkg	0.03988	-0.247
Agfg2	0.04260	-0.244
Ndufb5	0.03526	-0.244
Prxl2a	0.01638	-0.237
Pitpnm1	0.04720	-0.223
Ahcy	0.04338	-0.184
Atp5pd	0.03795	-0.174
L1cam	0.01534	-0.167
Actn1	0.01974	-0.083

B **RGS14 KO**

Upregulated after KA

Protein	Adj. p-value	Log2 FC (KA/Sal)
Spag7	0.00011	1.043
Srrm2	0.01341	0.755
Eif5b	0.03604	0.736
Cmip	0.02918	0.733
Mlst8	0.02592	0.705
Dnajb5	0.04264	0.697
Syt11	0.03861	0.626
Sez6l	0.00928	0.580
Trappc8	0.03487	0.549
Dlgap2	0.01553	0.495
Cnn3	0.03324	0.477
Scn1b	0.00837	0.430
Msi2	0.03794	0.407
Kif3a	0.02329	0.374
Tbca	0.03857	0.352
Celf2	0.03192	0.326
Sgta	0.00067	0.292
Ssr1	0.02485	0.289
Asb8	0.04264	0.277
Ggt7	0.03534	0.276
Slc27a4	0.01662	0.276
Farsb	0.02048	0.257
Tpt1	0.00416	0.237
Rps9	0.04456	0.237
Hsph1	0.01982	0.218
P4hb	0.02167	0.189
Eef2	0.00978	0.171

Downregulated after KA

Protein	Adj. p-value	Log2 FC (KA/Sal)
Ehbp1	0.02550	-1.389
Rnf141	0.04137	-0.896
Cacna1e	0.00274	-0.593
Ptgr2	0.01712	-0.461
Srprb	0.00560	-0.457
Mark3	0.01132	-0.422
Pmm1	0.00377	-0.402
Pip5k1a	0.01768	-0.397
Rgs7bp	0.00735	-0.391
Fth1	0.00248	-0.371
Mtmr1	0.01415	-0.363
Apoe	0.01321	-0.359
Hagh	0.03390	-0.324
Ncam1	0.03203	-0.318
Sorbs1	0.04719	-0.312
Mblac2	0.02923	-0.294
Mpp3	0.02482	-0.288
Abcb1a	0.03589	-0.285
Sh3kbp1	0.04989	-0.264
Scfd1	0.04290	-0.253
Xpnpep1	0.00664	-0.240
Sh3bgrl	0.03115	-0.168
Actr1b	0.03090	-0.148

Supplemental Table 2. Comprehensive list of differentially expressed proteins (DEPs) one day after KA-SE in WT or RGS14 KO hippocampi. (A) List of upregulated (left) or downregulated (right) DEPs one day following KA-SE in WT hippocampi, their adjusted p-values, and the fold change of abundance ($\log_2$ FC (KA/SAL)). (B) List of upregulated (left) or downregulated (right) DEPs one day following KA-SE in RGS14 KO hippocampi, their adjusted p-values, and the fold change of abundance ($\log_2$ FC (KA/SAL)). FC, fold change.
